# Supplementary material for: Structural and Biochemical Characterization of a Widespread Enterobacterial Peroxidase Encapsulin
Source: Adv Sci (Weinh). 2025 Apr 1;12(21):2415827. doi: 10.1002/advs.202415827 (PMC12140327; doi:10.1002/advs.202415827)
Supplement: Supplementary file 1 — Supporting Information [file ADVS-12-2415827-s001.pdf]

ADVANCED SCIENCE
Open Access

Supporting Information

for *Adv. Sci.*, DOI 10.1002/advs.202415827

Structural and Biochemical Characterization of a Widespread Enterobacterial Peroxidase Encapsulin

*Natalia C. Ubilla-Rodriguez, Michael P. Andreas and Tobias W. Giessen**

Supplementary Information

Structural and biochemical characterization of a widespread enterobacterial peroxidase encapsulin

Natalia C. Ubilla-Rodriguez[1], Michael P. Andreas[1], and Tobias W. Giessen[1]*
[1]Department of Biological Chemistry, University of Michigan Medical School, Ann Arbor, MI 48109, USA
*correspondence: tgiessen@umich.edu

Table of Contents

Supplementary Fig. 1 Comparison of staggered vs non-staggered co-expressions 2
Supplementary Fig. 2. Coomassie- and ferricyanide-stained Native-PAGE gels 3
Supplementary Fig. 3 TEM micrographs of Enc acid and peroxide exposure 4
Supplementary Fig. 4. Cryo-EM data processing workflow for EncDyP 5
Supplementary Fig. 5. Dimensions of heme cofactor 6
Supplementary Fig. 6. Analytical size exclusion chromatography (SEC) of free DyP 7
Supplementary Fig. 7. AlphaFold 3 prediction of a DyP subunit 8
Supplementary Fig. 8. Intrinsically disorder prediction of DyP 9
Supplementary Fig. 9. Details of TP-shell binding interaction 10
Supplementary Fig. 10. Cryo-EM data for the DyP hexamer 11
Supplementary Fig. 11. Cryo-EM data processing workflow for the DyP hexamer 12
Supplementary Fig. 12 List of organic peroxides used for activity assays 13
Supplementary Fig. 13. DyP heme access tunnel calculation 14
Supplementary Table 1. Saturation kinetics parameters of EncDyP and DyP 15
Supplementary Table 2. DNA sequences of constructs used in this study 16
Supplementary Table 3. Primers used in this study 20
Supplementary Table 4. Protein sequences of proteins used in this study 21
Supplementary Table 5. Cryo-EM data collection and refinement statistics 22
Supplementary References 23

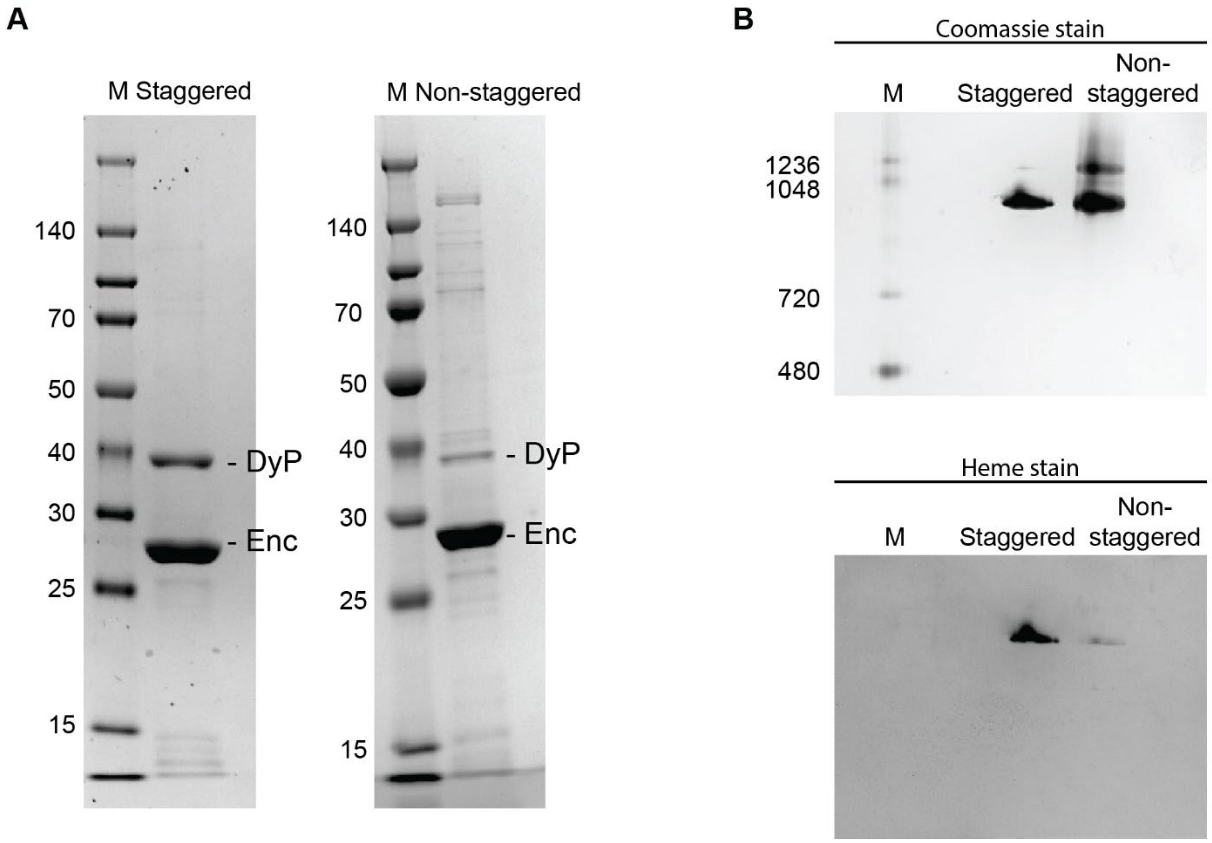

Supplementary Fig. 1. Comparison of staggered vs non-staggered expression strategies for EncDyP production. A) SDS-PAGE gels of purified EncDyP from staggered (left) and non-staggered (right) expressions. **B**) Native PAGE gel of purified EncDyP from staggered and non-staggered expressions stained for protein (top) and heme (bottom) highlighting the low heme loading in non-staggered expressions.

A

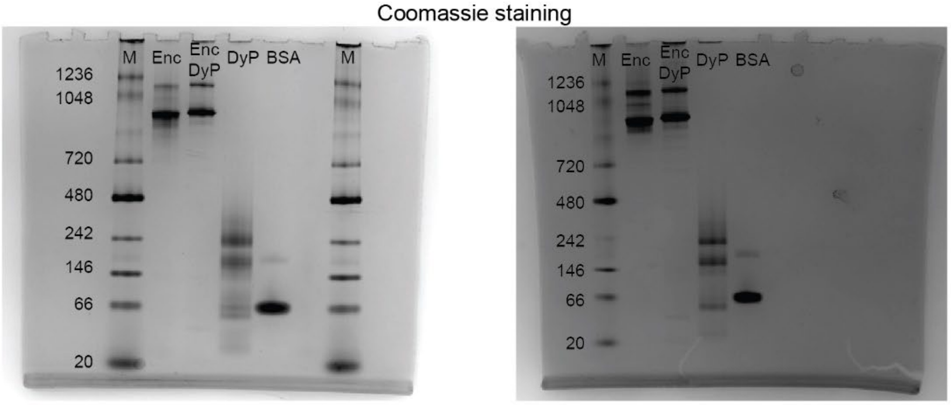

B

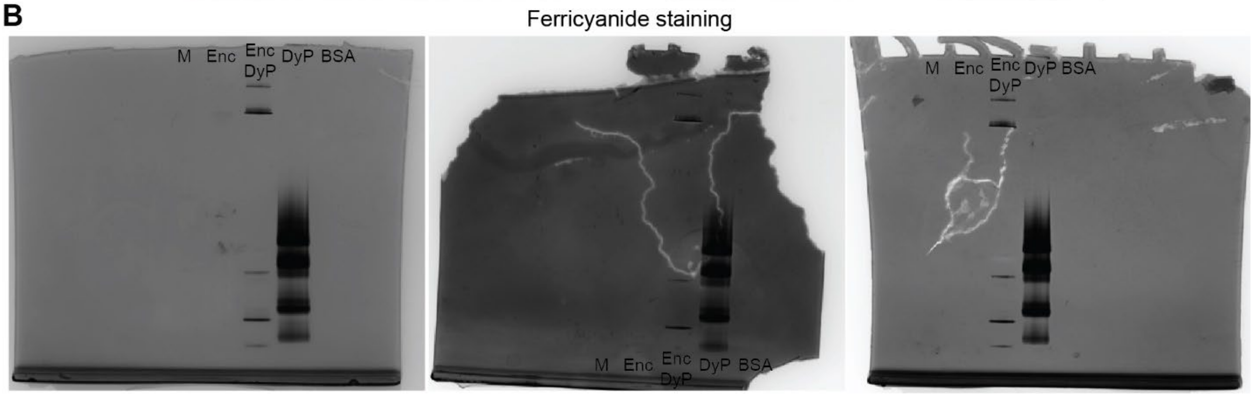

Supplementary Fig. 2. Uncropped Coomassie- and ferricyanide-stained Native-PAGE gels. **A**) Duplicate Coomassie-stained gels. **B**) Triplicate ferricyanide-stained gels.

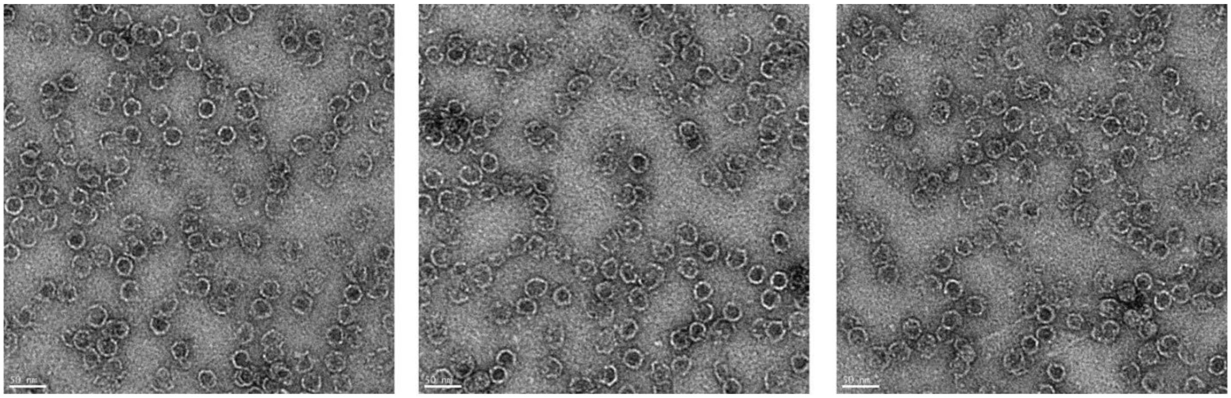

Supplementary Fig. 3. Additional TEM micrographs of Enc at pH 4.6 incubated with 9.8 mM H_2O_2 for 45 min. Scale bars: 50 nm.

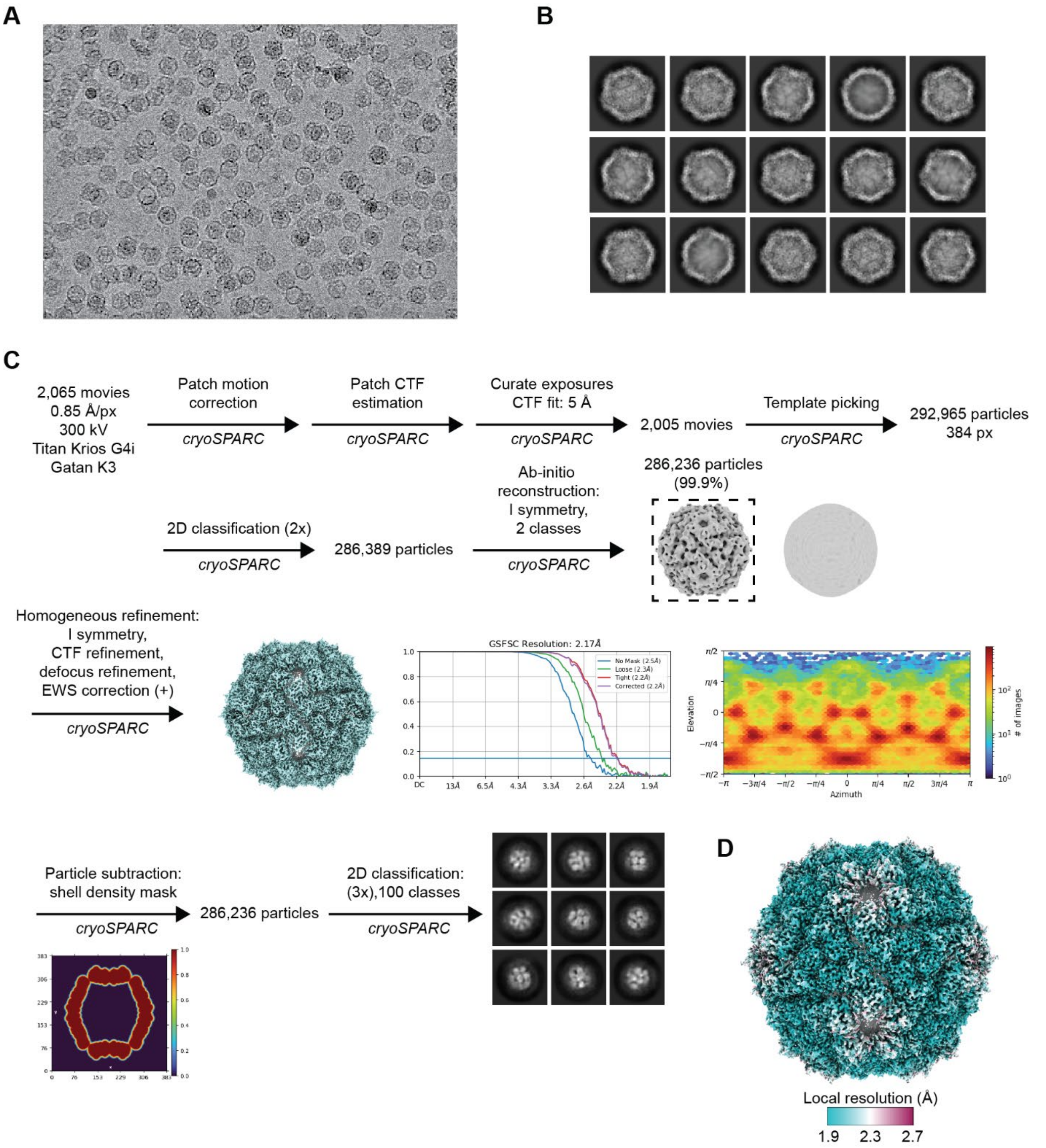

Supplementary Fig. 4. Cryo-EM data processing workflow for EncDyP. A) Representative raw micrograph. **B**) Representative 2D class averages. **C**) Cryo-EM data processing workflow. The global resolution estimate with FSC cut-off at 0.143 is shown. Angular distribution of particles used for final reconstruction is shown. Particle subtraction and 2D classification results of shell-subtracted particles is shown. **D**) Final map colored by local resolution.

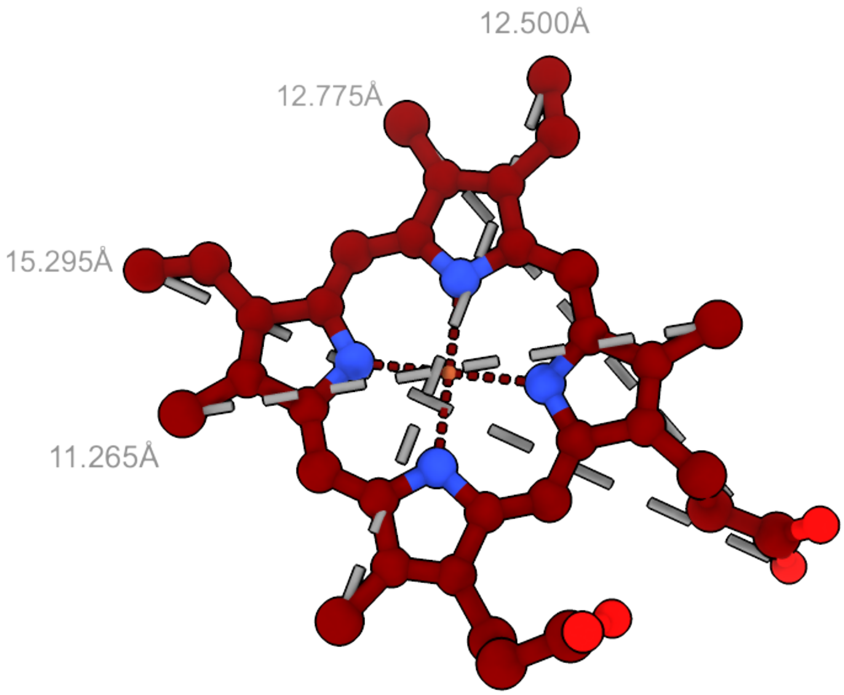

Supplementary Fig. 5. Dimensions of heme as measured in ChimeraX.

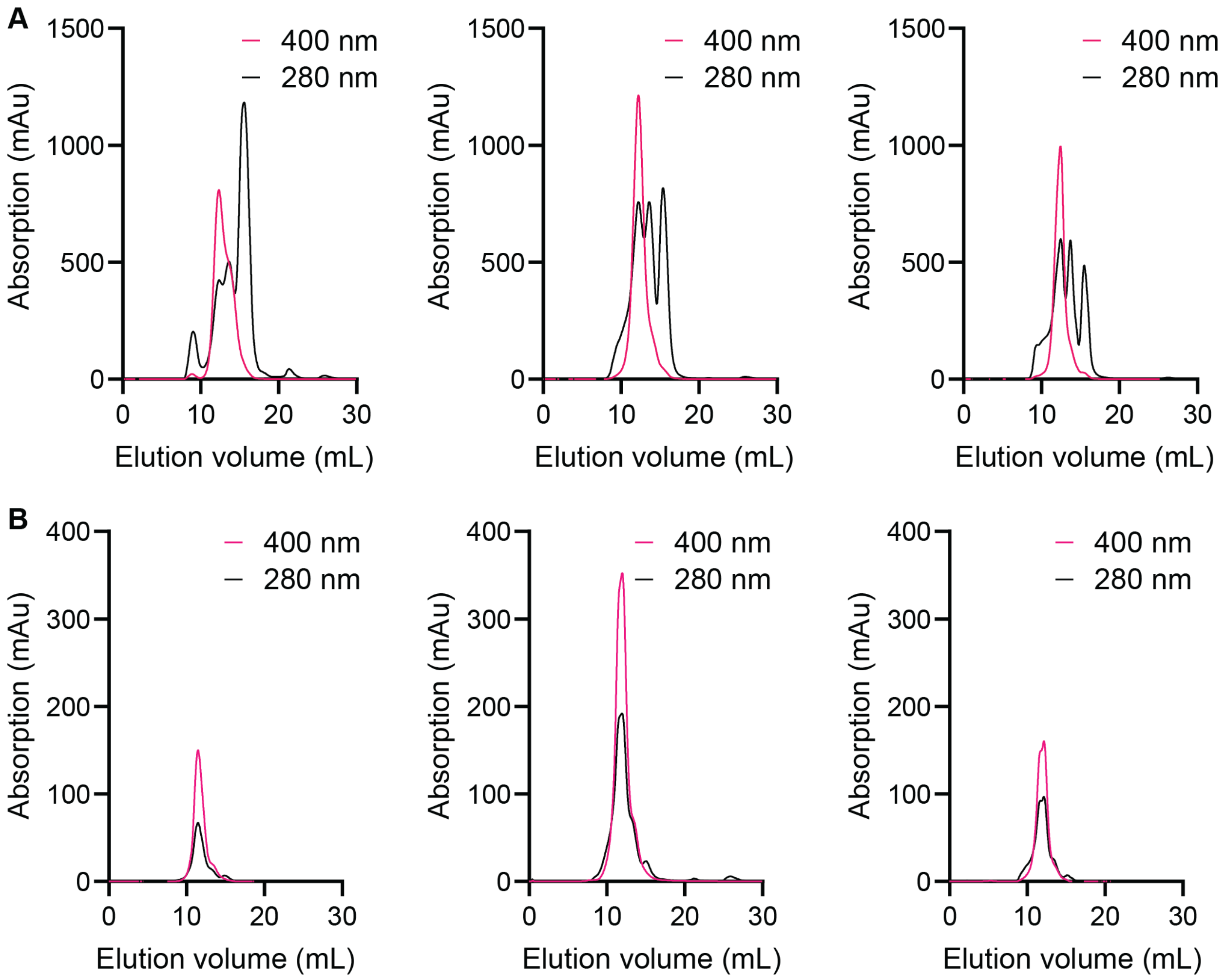

Supplementary Fig. 6. Analytical size exclusion chromatography (SEC) of free DyP. A) Analytical SEC triplicates of free DyP using a Superdex S-200 column. **B**) Analytical SEC triplicates of fraction 13 from A) using a Superdex S-200 column.

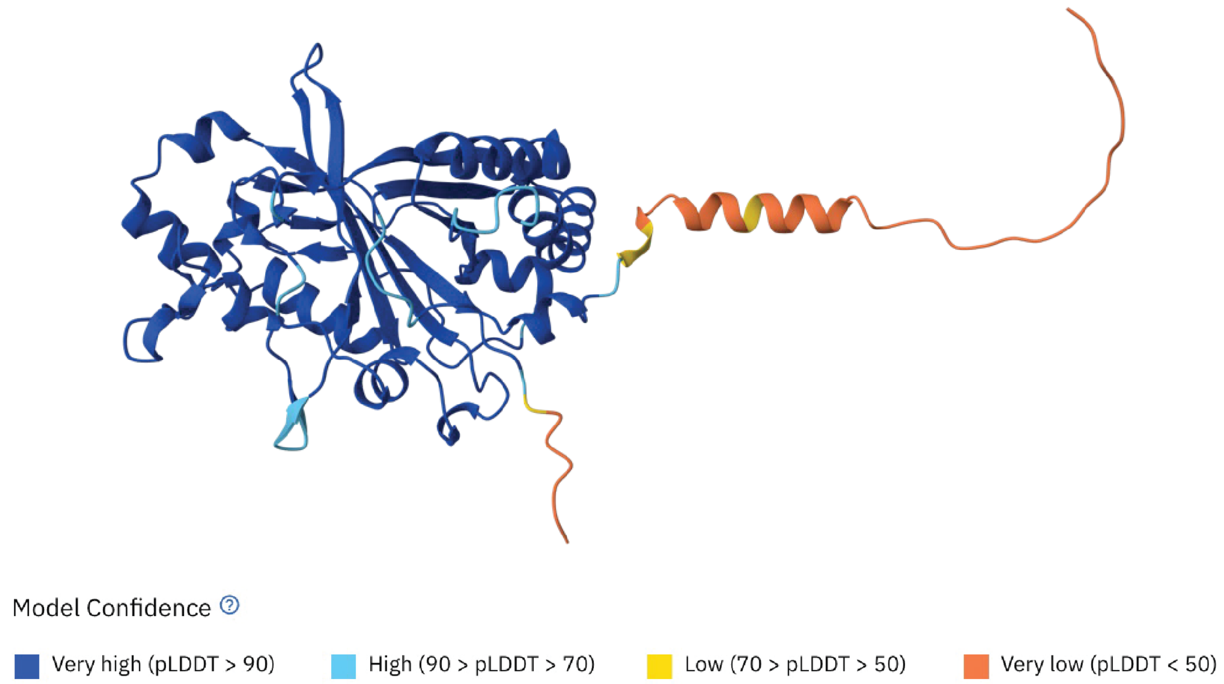

Supplementary Fig. 7. AlphaFold 3 prediction of a DyP subunit.[1, 2]

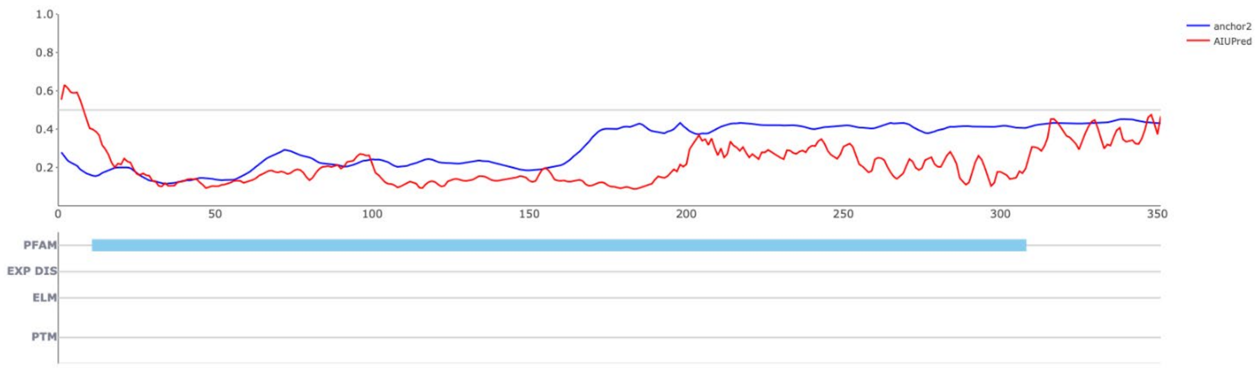

Supplementary Fig. 8. AIUPred-predicted intrinsically disordered regions of DyP based on the AIUPred and ANCHOR2 prediction algorithms.[3]

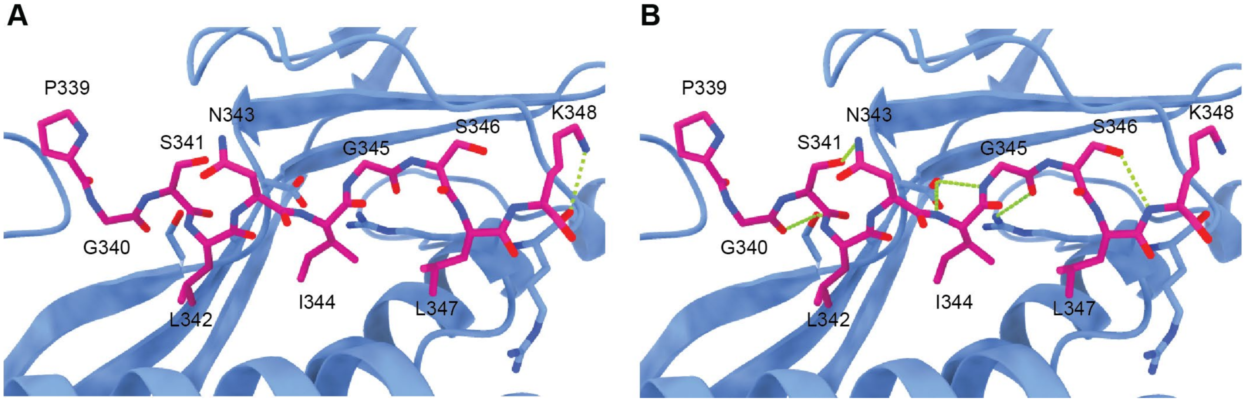

Supplementary Fig. 9. TP-shell binding is mediated by hydrogen-bonding and ionic interactions. A) Ionic interaction between the TP residue K348 and the Enc shell residue R35. **B**) Hydrogen bond network between the DyP TP and Enc shell. Two intramolecular and four intermolecular hydrogen bonds are highlighted.

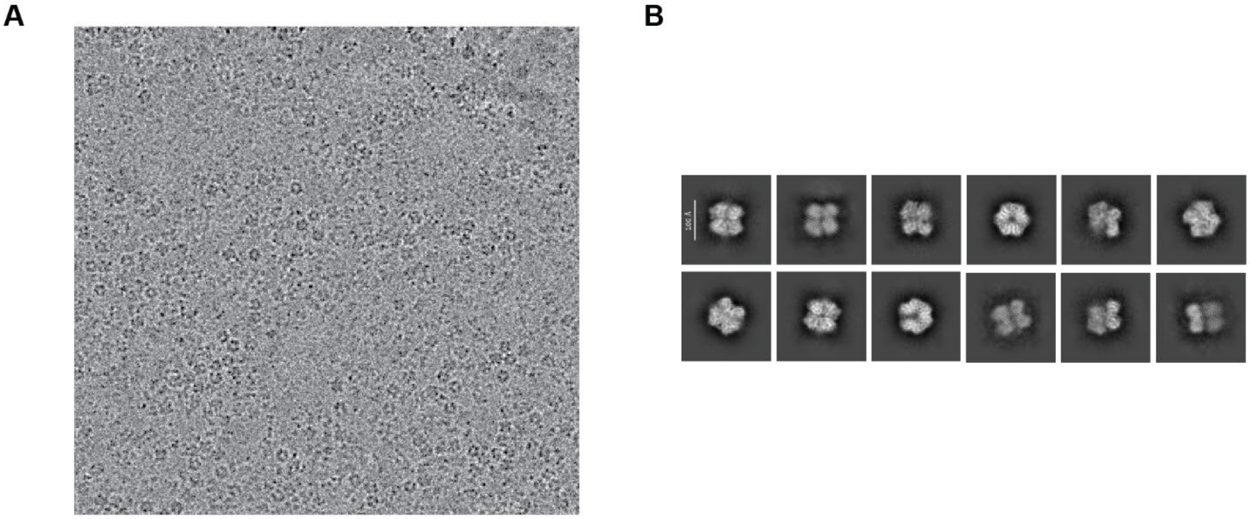

Supplementary Fig. 10. Cryo-EM data for the DyP hexamer. A) Representative raw micrograph. **B**) Representative 2D class averages.

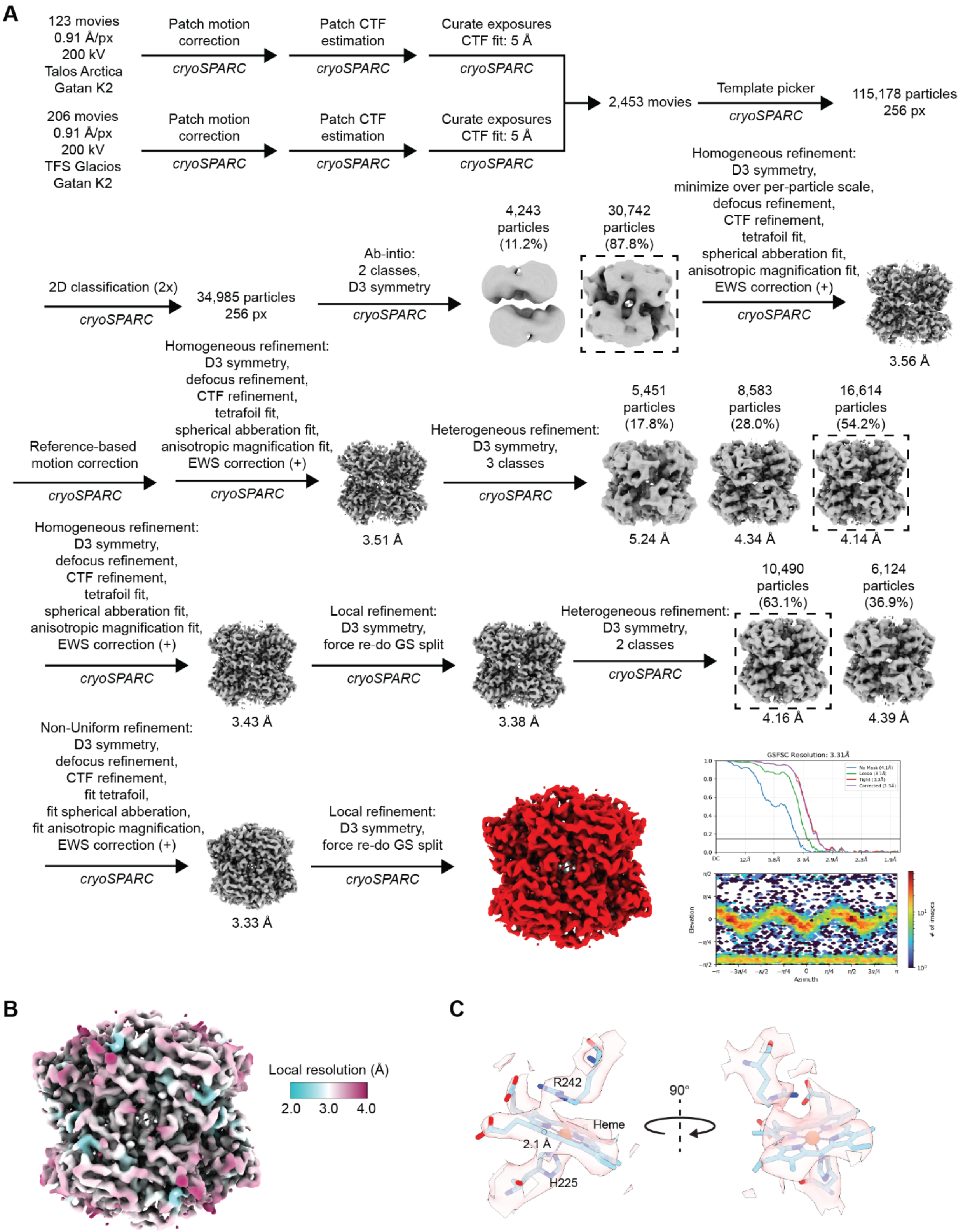

Supplementary Fig. 11. Cryo-EM data processing workflow for the DyP hexamer. A) Cryo-EM data processing workflow. The global resolution estimate with FSC cut-off at 0.143 is shown. Angular distribution of particles used for final reconstruction is shown. **B**) Final map colored by local resolution. **C**) Model and cryo-EM density for the heme active site.

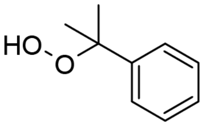

Cumene hydroperoxide
(CHP)

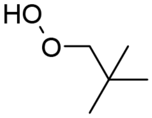

tert-butyl hydroperoxide
(TBHP)

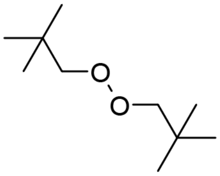

di-*tert*-butyl peroxide
(DTBP)

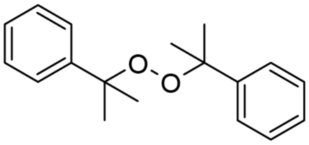

Dicumyl peroxide
(DCUP)

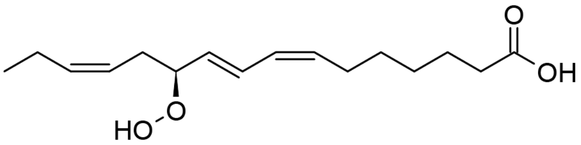

Hydroperoxyl-hexadecatrienoic acid
(HPHA)

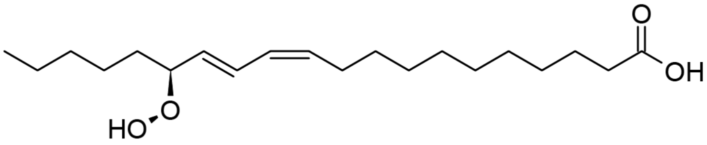

Hydroperoxyl-eicosadienoic acid
(HPEA)

Supplementary Fig. 12. Organic peroxide substrates tested for DyP activity with ABTS as the electron-donating substrate.

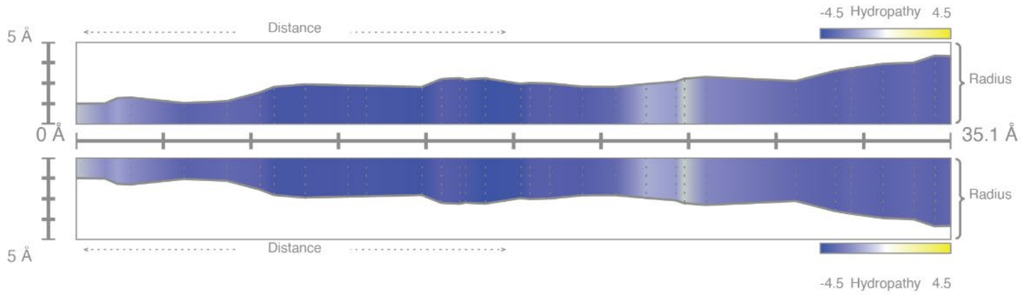

Supplementary Fig. 13. DyP heme access tunnel as computed using the MOLEonline server.[4]

Supplementary Table 1. Saturation kinetics parameters of EncDyP and DyP.

	**EncDyP**	**DyP**
v_{max} (µM s^{-1})	0.1962 ± 0.02	1.825 ± 0.12
K_m (µM)	63.85 ± 26.70	126.00 ± 36.95
k_{cat} (s^{-1})	1.308 ± 0.11	12.166 ± 0.81
k_{cat}/K_m (s^{-1} μM^{-1})	0.020 ± 0.01	0.096 ± 0.02

Supplementary Table 2. DNA sequences of constructs used in this study.

gBlock/construct	Plasmid	DNA sequence
DyP-Enc	pETDuet-1	ATGGGGTGTCCATTTTCACAGTCCGTCTCTCAACCTG<br>TGGATGAGAGACTTACGCGTGCAGCCATATTTCTGGT<br>TGTGACTATTAATCCCGGTAAAGCGGCTGAAGTCGCC<br>GTGCGTGCTCACTGCAGCATACTTTCCTCTCTTATCA<br>GAGGCGTCGGTTTTCGTATATCTGATGGTGGGTTGTC<br>GTGTGTCATGGGGGTGAGTGAAGGAGGGTGGGAGC<br>GCCTTTTTGGGGATACAAAACCTGAATATTTGCATGTA<br>TTCCGTGAAATCAATGGTGTACATCATGCTCCATCAAC<br>ACCCGGAGACCTGTTATATCATATTCGCGCCGCCCGG<br>ATGGATCTTTGTTTTGAGCTGGCCAGCCGGATACTGT<br>CGGATCTCGGCAACTCGGTTAGTGTTGTTGACTCCGT<br>TCAGGGTTTTCGGTATTTCGACGATCGCGATCTATTAG<br>GTTTTGTTGACGGTACTGAAAATCCGGTCGCTCAGGC<br>CGCTGTGGATGCAACCCTGATTGGTGATGAGGACAT<br>GGTATTCGCTGGCGGGAGCTATGTGATCGTCCAGAA<br>ATACCTGCATGATCTTGATAAATGGAATGCGATTCCTG<br>TTGAGCAGCAGGAAAAGATTATTGGCCGCGAAAAACT<br>ATCAGACATTGAGCTCAGGGATGCTGATAAACCCAGT<br>TATGCTCATAATGTACTCACCAGTATTGAAGAGGATGG<br>CGAGGACGTCGACATACTGCGTGACAATATGCCATTT<br>GGCGATCCCGGCAAAGGCGAGTTCGGAACCTATTTT<br>ATCGGTTACTCCCGAAAACCTGAACGTATTGAAAGGA<br>TGCTTGAGAATATGTTTATTGGTAATCCACCGGGTAAT<br>TATGATCGGATCCTGGATGTCAGCCGTGCGATAACGG<br>GAACATTATTTTTCGTTCCGACGACCAGCTTCCTGGA<br>CAGTATTGAACCCCAGTCAGCTCCCGGTCAACAGGG<br>AGATGATGTGATAAATACACTACGCTCAACCGCCATTA<br>AAGGAGACATAATGCCCGGTTCCCTTAATATTGGTTC<br>GTTAAAAAAAGAGGTTTAGAATGAATAATCTACATCGC<br>GAACTTGCGCCTGTTTCCGATGCGGCGTGGGAACAG<br>ATTGAAGAGGAAGCAACCCGTACACTTAAACGTTTCC<br>TTGCTGCTCGGCGGGTGGTGGATGTCACTGATCCTC<br>AGGGGGCGGCATTGTCGGCTGTTGGGACAGGTCAT<br>GTTGCCTATCTGGATGGCCCCTGTGCAGGGGTAAGT<br>GCAGTAAAACGTCAGGTACTGCCGGTGGTTGAATTCA<br>GGGTCCCGTTTAAATTAACTCGTCAGGCAATTGATGA<br>TGTTGAACGTGGCTCTCAGGATTCGGACTGGTCCCC<br>TCTAAAAGAAGCTGCCCGCAAAATTGCGGCTGCAGA<br>GGACCAGACAATTTTTGATGGCTACATGGCGGCAGG<br>TATTGCTGGGATTCGACCACAGTCGAGTAATACACCT<br>TTAACCTTACCTGCTACTGCTTCAGATTATCCAACGGT<br>GGTTGCTCAGGCACTGGATCAGTTACGCGTGGCAGG<br>AGTGAATGGTCCCTACCATCTGGTGTTAGGTGAAAAG<br>GCTTATACGTCGATTACCGGCGGTAATGAAGGCGGTT<br>ATCCGGTATTCCAGCATATCCGCCGGCTTATCGATGG<br>TGAAATTGTCTGGGCCCCTGCTATCGAAGGTGGATTA<br>CTGTTAACCACTCGTGGCGGTGATTTTGTCATGGATA<br>TCGGCCAAGATATATCCATAGGTTACCTGAACCATACC<br>GGCACAGATGTTGAGCTGTATCTGCAGGAAAGTTTCA

		CTTTCAGTGCACTCACGTCAGAAGCCACAGTAACATT<br>ACTTCCTCCTGAAGAATGA
His_6-GGS-TEV-GGS-DyP	pETDuet-1	ATGCACCATCACCATCACCATGGCGGCAGCGAGAAT<br>CTGTATTTTCAGAGCGGCGGCAGCGGGTGTCCATTTT<br>CACAGTCCGTCTCTCAACCTGTGGATGAGAGACTTAC<br>GCGTGCAGCCATATTTCTGGTTGTGACTATTAATCCCG<br>GTAAAGCGGCTGAAGTCGCCGTGCGTGCTCACTGCA<br>GCATACTTTCCTCTCTTATCAGAGGCGTCGGTTTTCG<br>TATATCTGATGGTGGGTTGTCGTGTGTCATGGGGGTG<br>AGTGAAGGAGGGTGGGAGCGCCTTTTTGGGGATACA<br>AAACCTGAATATTTGCATGTATTCCGTGAAATCAATGG<br>TGTACATCATGCTCCATCAACACCCGGAGACCTGTTA<br>TATCATATTCGCGCCGCCCGGATGGATCTTTGTTTTGA<br>GCTGGCCAGCCGGATACTGTCGGATCTCGGCAACTC<br>GGTTAGTGTTGTTGACTCCGTTCAGGGTTTTCGGTAT<br>TTCGACGATCGCGATCTATTAGGTTTTGTTGACGGTA<br>CTGAAAATCCGGTCGCTCAGGCCGCTGTGGATGCAA<br>CCCTGATTGGTGATGAGGACATGGTATTCGCTGGCG<br>GGAGCTATGTGATCGTCCAGAAATACCTGCATGATCT<br>TGATAAATGGAATGCGATTCCTGTTGAGCAGCAGGAA<br>AAGATTATTGGCCGCGAAAAACTATCAGACATTGAGC<br>TCAGGGATGCTGATAAACCCAGTTATGCTCATAATGTA<br>CTCACCAGTATTGAAGAGGATGGCGAGGACGTCGAC<br>ATACTGCGTGACAATATGCCATTTGGCGATCCCGGCA<br>AAGGCGAGTTCGGAACCTATTTTATCGGTTACTCCCG<br>AAAACCTGAACGTATTGAAAGGATGCTTGAGAATATGT<br>TTATTGGTAATCCACCGGGTAATTATGATCGGATCCTG<br>GATGTCAGCCGTGCGATAACGGGAACATTATTTTTCG<br>TTCCGACGACCAGCTTCCTGGACAGTATTGAACCCCA<br>GTCAGCTCCCGGTCAACAGGGAGATGATGTGATAAAT<br>ACACTACGCTCAACCGCCATTAAAGGAGACATAATGC<br>CCGGTTCCCTTAATATTGGTTCGTTAAAAAAAGAGGTT<br>TAG
Enc	pCDFDuet1	ATGAATAATCTACATCGCGAACTTGCGCCTGTTTCCG<br>ATGCGGCGTGGGAACAGATTGAAGAGGAAGCAACCC<br>GTACACTTAAACGTTTCCTTGCTGCTCGGCGGGTGGT<br>GGATGTCACTGATCCTCAGGGGGCGGCATTGTCGGC<br>TGTTGGGACAGGTCATGTTGCCTATCTGGATGGCCCC<br>TGTGCAGGGGTAAGTGCAGTAAAACGTCAGGTACTG<br>CCGGTGGTTGAATTCAGGGTCCCGTTTAAATTAACTC<br>GTCAGGCAATTGATGATGTTGAACGTGGCTCTCAGGA<br>TTCGGACTGGTCCCCTCTAAAAGAAGCTGCCCGCAA<br>AATTGCGGCTGCAGAGGACCAGACAATTTTTGATGGC<br>TACATGGCGGCAGGTATTGCTGGGATTCGACCACAGT<br>CGAGTAATACACCTTTAACCTTACCTGCTACTGCTTCA<br>GATTATCCAACGGTGGTTGCTCAGGCACTGGATCAGT<br>TACGCGTGGCAGGAGTGAATGGTCCCTACCATCTGG<br>TGTTAGGTGAAAAGGCTTATACGTCGATTACCGGCGG<br>TAATGAAGGCGGTTATCCGGTATTCCAGCATATCCGC<br>CGGCTTATCGATGGTGAAATTGTCTGGGCCCCTGCTA<br>TCGAAGGTGGATTACTGTTAACCACTCGTGGCGGTG

		ATTTTGTCATGGATATCGGCCAAGATATATCCATAGGTT<br>ACCTGAACCATACCGGCACAGATGTTGAGCTGTATCT<br>GCAGGAAAGTTTCACTTTCAGTGCACTCACGTCAGAA<br>GCCACAGTAACATTACTTCCTCCTGAAGAATGA
DyP	pCDFDuet-1	ATGGGGTGTCCATTTTCACAGTCCGTCTCTCAACCTG<br>TGGATGAGAGACTTACGCGTGCAGCCATATTTCTGGT<br>TGTGACTATTAATCCCGGTAAAGCGGCTGAAGTCGCC<br>GTGCGTGCTCACTGCAGCATACTTTCCTCTCTTATCA<br>GAGGCGTCGGTTTTCGTATATCTGATGGTGGGTTGTC<br>GTGTGTCATGGGGGTGAGTGAAGGAGGGTGGGAGC<br>GCCTTTTTGGGGATACAAAACCTGAATATTTGCATGTA<br>TTCCGTGAAATCAATGGTGTACATCATGCTCCATCAAC<br>ACCCGGAGACCTGTTATATCATATTCGCGCCGCCCGG<br>ATGGATCTTTGTTTTGAGCTGGCCAGCCGGATACTGT<br>CGGATCTCGGCAACTCGGTTAGTGTTGTTGACTCCGT<br>TCAGGGTTTTCGGTATTTCGACGATCGCGATCTATTAG<br>GTTTTGTTGACGGTACTGAAAATCCGGTCGCTCAGGC<br>CGCTGTGGATGCAACCCTGATTGGTGATGAGGACAT<br>GGTATTCGCTGGCGGGAGCTATGTGATCGTCCAGAA<br>ATACCTGCATGATCTTGATAAATGGAATGCGATTCCTG<br>TTGAGCAGCAGGAAAAGATTATTGGCCGCGAAAAACT<br>ATCAGACATTGAGCTCAGGGATGCTGATAAACCCAGT<br>TATGCTCATAATGTACTCACCAGTATTGAAGAGGATGG<br>CGAGGACGTCGACATACTGCGTGACAATATGCCATTT<br>GGCGATCCCGGCAAAGGCGAGTTCGGAACCTATTTT<br>ATCGGTTACTCCCGAAAACCTGAACGTATTGAAAGGA<br>TGCTTGAGAATATGTTTATTGGTAATCCACCGGGTAAT<br>TATGATCGGATCCTGGATGTCAGCCGTGCGATAACGG<br>GAACATTATTTTTCGTTCCGACGACCAGCTTCCTGGA<br>CAGTATTGAACCCCAGTCAGCTCCCGGTCAACAGGG<br>AGATGATGTGATAAATACACTACGCTCAACCGCCATTA<br>AAGGAGACATAATGCCCGGTTCCCTTAATATTGGTTC<br>GTTAAAAAAAGAGGTTTAG
Enc	pBAD/HisA	ATGAATAATCTACATCGCGAACTTGCGCCTGTTTCCG<br>ATGCGGCGTGGGAACAGATTGAAGAGGAAGCAACCC<br>GTACACTTAAACGTTTCCTTGCTGCTCGGCGGGTGGT<br>GGATGTCACTGATCCTCAGGGGGCGGCATTGTCGGC<br>TGTTGGGACAGGTCATGTTGCCTATCTGGATGGCCCC<br>TGTGCAGGGGTAAGTGCAGTAAAACGTCAGGTACTG<br>CCGGTGGTTGAATTCAGGGTCCCGTTTAAATTAACTC<br>GTCAGGCAATTGATGATGTTGAACGTGGCTCTCAGGA<br>TTCGGACTGGTCCCCTCTAAAAGAAGCTGCCCGCAA<br>AATTGCGGCTGCAGAGGACCAGACAATTTTTGATGGC<br>TACATGGCGGCAGGTATTGCTGGGATTCGACCACAGT<br>CGAGTAATACACCTTTAACCTTACCTGCTACTGCTTCA<br>GATTATCCAACGGTGGTTGCTCAGGCACTGGATCAGT<br>TACGCGTGGCAGGAGTGAATGGTCCCTACCATCTGG<br>TGTTAGGTGAAAAGGCTTATACGTCGATTACCGGCGG<br>TAATGAAGGCGGTTATCCGGTATTCCAGCATATCCGC<br>CGGCTTATCGATGGTGAAATTGTCTGGGCCCCTGCTA<br>TCGAAGGTGGATTACTGTTAACCACTCGTGGCGGTG

		ATTTTGTCATGGATATCGGCCAAGATATATCCATAGGTTACCTGAACCATACCGGCACAGATGTTGAGCTGTATCTGCAGGAAAGTTTCACTTTCAGTGCACTCACGTCAGAAGCCACAGTAACATTACTTCCTCCTGAAGAATGA

Supplementary Table 3. Primers used in this study.

Primer	DNA sequence
NU1 F	AAGTATAAGAAGGAGATATACAATGGGGTGTCCATTTTCACAGT
NU1 R	GCAGCAGCCTAGGTTAATTCATTCTTCAGGAGGAAGTAATGT
NU5 F	AAGTATAAGAAGGAGATATACAATGCACCATCACCATCACCA
NU5 R	TGGACACCCGCTGCCGCCGCTCTGAAAAT
NU8 F	AAGTATAAGAAGGAGATATACAATGAATAATCTACATCGCGAACTTGC
NU8 R	GCGGTTTCTTTACCAGACTCATTCTTCAGGAGGAAGTAATGT
NU10 F	AAGTATAAGAAGGAGATATACAATGGGGTGTCCATTTTCACAGT
NU10 R	GCGGTTTCTTTACCAGACCTAAACCTCTTTTTTTAACGAACCA
NU11 F	GCTAACAGGAGGAATTAACATGAATAATCTACATCGCGAACTTGC
NU11 R	CAAAACAGCCAAGCTTCGTCATTCTTCAGGAGGAAGTAATGT

Supplementary Table 4. Protein sequences of proteins used in this study.

Protein	Protein sequence
Enc	MNNLHRELAPVSDAAWEQIEEEATRTLKRFLAARRVVDVTDPQGAALSAVGTGHVAYLDGPCAGVSAVKRQVLPVVEFRVPFKLTRQAIDDVERGSQDSDWSPLKEAARKIAAAEDQTIFDGYMAAGIAGIRPQSSNTPLTLPATASDYPTVVAQALDQLRVAGVNGPYHLVLGEKAYTSITGGNEGGYPVFQHIRRLIDGEIVWAPAIEGGLLLTTRGGDFVMDIGQDISIGYLNHTGTDVELYLQESFTFSALTSEATVTLLPPEE
DyP	MGCPFSQSVSQPVDERLTRAAIFLVVTINPGKAAEVAVRAHCSILSSLIRGVGFRISDGGLSCVMGVSEGGWERLFGDTKPEYLHVFREINGVHHAPSTPGDLLYHIRAARMDLCFELASRILSDLGNSVSVVDSVQGFRYFDDRDLLGFVDGTENPVAQAAVDATLIGDEDMVFAGGSYVIVQKYLHDLDKWNAIPVEQQEKIIGREKLSDIELRDADKPSYAHNVLTSIEEDGEDVDILRDNMPFGDPGKGEFGTYFIGYSRKPERIERMLENMFIGNPPGNYDRILDVSRAITGTLFFVPTTSFLDSIEPQSAPGQQGDDVINTLRSTAIKGDIMPGSLNIGSLKKEV
His_6-GGS-TEV-GGS-DyP	MHHHHHHGGSENLYFQSGGSGCPFSQSVSQPVDERLTRAAIFLVVTINPGKAAEVAVRAHCSILSSLIRGVGFRISDGGLSCVMGVSEGGWERLFGDTKPEYLHVFREINGVHHAPSTPGDLLYHIRAARMDLCFELASRILSDLGNSVSVVDSVQGFRYFDDRDLLGFVDGTENPVAQAAVDATLIGDEDMVFAGGSYVIVQKYLHDLDKWNAIPVEQQEKIIGREKLSDIELRDADKPSYAHNVLTSIEEDGEDVDILRDNMPFGDPGKGEFGTYFIGYSRKPERIERMLENMFIGNPPGNYDRILDVSRAITGTLFFVPTTSFLDSIEPQSAPGQQGDDVINTLRSTAIKGDIMPGSLNIGSLKKEV

Supplementary Table 5. Cryo-EM data collection and refinement statistics.

	EncDyP (EMD-47525) (PDB 9E5E)	DyP (EMD-47518) (PDB 9E4R)
**Data collection and processing**		
Magnification	105,000x	45,000x
Voltage (kV)	300	200
Electron exposure ($e^-/Å^2$)	39.91	46.24 and 47.84
Defocus range (mm)	-0.8 to -2.5	-1.0 to -1.8
Pixel size (Å)	0.8487	0.91
Symmetry imposed	I	D3
Initial particle images (no.)	292,965	115,187
Final particle images (no.)	286,236	10,490
Map resolution (Å)	2.17	3.31
FSC threshold	0.143	0.143
**Refinement**		
Initial model used (PDB code)	AlphaFold	AlphaFold
Model resolution (Å)	2.4	3.5
FSC threshold	0.5	0.5
Map sharpening *B* factor ($Å^2$)	-85.5	-105.9
Model composition		
Non-hydrogen atoms	2091	2,464
Protein residues	277	311
Ligands	-	1
B factors ($Å^2$)		
Protein	31.26	85.09
Ligands	-	50.95
r.m.s. deviations		
Bond lengths (Å)	0.004	0.004
Bond angles (°)	1.049	0.998
Validation		
MolProbity score	1.07	1.44
Clashscore	2.40	5.16
Poor rotamers (%)	0.45	0.38
Ramachandran plot		
Favored (%)	97.8	97.09
Allowed (%)	2.2	2.91
Disallowed (%)	0	0

References

(1) Jumper, J.; Evans, R.; Pritzel, A.; Green, T.; Figurnov, M.; Ronneberger, O.; Tunyasuvunakool, K.; Bates, R.; Žídek, A.; Potapenko, A. Highly accurate protein structure prediction with AlphaFold. *nature* **2021**, *596* (7873), 583-589.
(2) Varadi, M.; Bertoni, D.; Magana, P.; Paramval, U.; Pidruchna, I.; Radhakrishnan, M.; Tsenkov, M.; Nair, S.; Mirdita, M.; Yeo, J. AlphaFold Protein Structure Database in 2024: providing structure coverage for over 214 million protein sequences. *Nucleic acids research* **2024**, *52* (D1), D368-D375.
(3) Erdős, G.; Dosztányi, Z. AIUPred: combining energy estimation with deep learning for the enhanced prediction of protein disorder. *Nucleic Acids Research* **2024**, gkae385.
(4) Pravda, L.; Sehnal, D.; Toušek, D.; Navrátilová, V.; Bazgier, V.; Berka, K.; Svobodová Vařeková, R.; Koča, J.; Otyepka, M. MOLEonline: a web-based tool for analyzing channels, tunnels and pores (2018 update). *Nucleic acids research* **2018**, *46* (W1), W368-W373.
